# PD-L1 biomarker testing for non-small cell lung cancer: truth or fiction?

**DOI:** 10.1186/s40425-016-0153-x

**Published:** 2016-08-16

**Authors:** Claud Grigg, Naiyer A. Rizvi

**Affiliations:** NewYork-Presbyterian/Columbia University Medical Center, Hematology/Oncology, 177 Fort Washington Avenue, 6GN-435, New York, NY 10032 USA

**Keywords:** NSCLC, PD-1, PD-L1, Immunotherapy, Nivolumab, Pembrolizumab, Immune checkpoint inhibitor, Biomarker, Lung cancer

## Abstract

Research in cancer immunology is currently accelerating following a series of cancer immunotherapy breakthroughs during the last 5 years. Various monoclonal antibodies which block the interaction between checkpoint molecules PD-1 on immune cells and PD-L1 on cancer cells have been used to successfully treat non-small cell lung cancer (NSCLC), including some durable responses lasting years. Two drugs, nivolumab and pembrolizumab, are now FDA approved for use in certain patients who have failed or progressed on platinum-based or targeted therapies while agents targeting PD-L1, atezolizumab and durvalumab, are approaching the final stages of clinical testing. Despite impressive treatment outcomes in a subset of patients who receive these immune therapies, many patients with NSCLC fail to respond to anti-PD-1/PD-L1 and the identification of a biomarker to select these patients remains highly sought after. In this review, we discuss the recent clinical trial results of pembrolizumab, nivolumab, and atezolizumab for NSCLC, and the significance of companion diagnostic testing for tumor PD-L1 expression.

## Background

Non-small cell lung cancer (NSCLC) is by far the leading cause of cancer related mortality in the US and worldwide [1, 2]. It is frequently diagnosed in the metastatic or unresectable setting, while those patients that do undergo potentially curative surgery will frequently relapse [3]. Despite advances in cancer treatment and survival over the last 30 years, improvements in survival for lung cancer patients have been comparatively modest, prompting research into new modalities of therapy for lung cancer [2]. The introduction of molecularly targeted agents to NSCLC therapy was a major breakthrough, though these drugs benefit only a small proportion of patients (mostly never smokers) who harbor activating genetic alterations including EGFR, ALK, and ROS1 [4].

NSCLC was not traditionally considered to be an immunotherapy responsive tumor type when the earliest clinical trials employed interleukin-2, vaccines, and interferons [5]. More recently, major treatment responses have been observed with the use of immune checkpoint inhibitors. Immune checkpoints are proteins on the surface of lymphocytes and other immune cells, most notably on cytotoxic T-cells. When bound to their specific ligand, often another surface bound protein on a neighboring cell, they can transmit stimulatory or inhibitory signals to activate or dampen the cellular adaptive immune response [6]. Mounting evidence suggests that the predominant mechanism by which NSCLC evades detection and elimination by the immune system is by exploiting one such inhibitory pathway through the expression of programmed death ligand 1 (PD-L1, B7-H1) [7]. PD-L1 then binds to its receptor, programmed cell death protein 1 (PD-1), on surveilling lymphocytes and initiates a signaling cascade which leads to lymphocyte exhaustion, a state of impaired function [8].

The most successful immune checkpoint inhibitors so far are monoclonal antibodies which bind to either PD-1 or PD-L1 and prevent their interaction at the tumor-immune interface. The depth and durability of responses in NSCLC have revolutionized the conceptual approach to lung cancer treatment. Still, fewer than a quarter to half of patients, even in highly selected cohorts, have experienced a clinical benefit while on anti-PD-1 or anti-PD-L1 therapies. In this review, we will describe the recent data leading to the FDA approvals of nivolumab (Opdivo, approved in December 2014 and March 2015 for non-squamous and squamous NSCLC in the 2^nd^ line setting) and pembrolizumab (Keytruda, approved in October 2015 for PD-L1 positive NSCLC also in the 2^nd^ line setting). We will also explore the ongoing effort and challenges to identify a PD-L1 assay that can select patients who will benefit from these drugs.

### Nivolumab

Four large clinical trials have reported on the use of nivolumab for NSCLC, however some of the most important observations came from the initial phase I trial published in 2010. That trial enrolled patients with diverse malignancies, including six patients with NSCLC, but only a small number of responses were seen [9]. In several patients, pre- and post- treatment biopsies were tested for PD-L1 expression by immunohistochemistry (IHC) using a non-commercial antibody (5H1), with an indication that PD-L1 expression correlated with response. Post-treatment tumor lesions were infiltrated with CD8+ but not CD4+ lymphocytes. In the peripheral blood, T-cell markers, but not B- or NK-cell markers, were noted to drop by day 2 of treatment, but then increased substantially and remained elevated for about 30 days. These findings suggested that the effects of nivolumab treatment were rapid and resulted in a major redistribution of existing lymphocytes, followed by a prolonged period of immune activation which correlated with the anti-tumor response [9].

A much larger phase I study was reported in 2012, which included 122 evaluable patients with heavily pre-treated NSCLC [10]. Treatment was well tolerated, though fatigue was common and seen in approximately 40 % of patients who experienced a treatment related adverse event (AE). Serious AEs occurred in 11 % of all patients including two deaths in the NSCLC cohort due to pneumonitis. These early deaths may have been preventable with earlier intervention, as the autoimmune toxicities of the drugs were still poorly understood at the time. Still, just 5 % of patients discontinued treatment for toxicity reasons and some patients with immune-related endocrinopathies, colon and liver toxicities tolerated treatment re-challenge. Clinical activity was evident, with 14 responses (18 %) seen in the NSCLC group, and 3 mg/kg (every 2 weeks) was identified as the optimal dose. Of 61 pre-treatment tumors in the study tested for PD-L1 using the same 5H1 antibody, 36 % of positive patients but none of the negative patients responded [10].

Long-term follow-up is now available from the phase I expansion, totaling 129 NSCLC patients with a median follow-up of 39 months (up to 66 months) as of 2015 [11]. All patients had failed at least one chemotherapy regimen, 54 % had failed three or more. The overall response rate was 17 % (and was 22 % at the doses considered therapeutic: 3 mg/kg and 10 mg/kg) with most responses lasting over a year and the longest ongoing at 3 years. A further 5 % of patients experienced an initial disease pseudo-progression or a mixed response, both phenomena consistent with an immune pattern of response that is not captured by standard Response Evaluation Criteria in Solid Tumors (RECIST) criteria [12]. Responses were seen regardless of subgroups: squamous and non-squamous, EGFR and KRAS mutated, PD-L1 positive and negative, while those with a smoking history more than 5 pack-years did much better (overall response rate [ORR] 30 % vs 0 for <5 pack-years). The median overall survival (OS) was 9.9 months, and at 3 years 27 % of patients were still alive and 9 % progression-free. Only six treatment related grade 3 or 4 AEs were reported including three pneumonitis, one colitis, and one hepatitis. One reassuring observation was that half of the 18 patients who discontinued therapy for toxicity had a continued response lasting > 9 months, suggesting that early re-challenging with anti-PD-1 therapies after toxicity may not be necessary in some patients who have responded.

Three phase II and III clinical trials were initiated to evaluate squamous and non-squamous tumors independently. For squamous NSCLC, a phase II single arm study enrolled 117 heavily pre-treated patients with unresectable disease, the vast majority of whom were prior or current smokers [13]. The best ORR was 14.5 %, with one patient having a complete response by investigator assessment. Treatment toxicity was higher in this population, 27 % of patients required a dose delay and 17 % experienced a treatment related grade 3 or 4 AE, including four patients with pneumonitis and three with colitis. Low grade gastrointestinal (GI) toxicity and fatigue were also common but manageable.

CHECKMATE 017 and CHECKMATE 057 were parallel, randomized controlled phase III trials for advanced, platinum-refractory squamous and non-squamous NSCLC, respectively, and are summarized in Table 1. In CHECKMATE 017 [14], nivolumab bested docetaxel (75 mg/m^2^ every 3 weeks) in terms of the primary endpoint OS (median 9.2 vs 6.0 months; hazard ratio [HR] 0.59, *p* < 0.001) and ORR (20 % vs 9 %; odds ratio [OR] 2.6, *p* = 0.008). It is important to mention that 34 % of patients had previously received paclitaxel, though this is unlikely to fully account for the differential activities. Consistent with the earlier studies, 9 patients on nivolumab (6.5 %) experienced a delayed response following an initial pseudo-progression. Nivolumab was much better tolerated than docetaxel, with grade 3 or 4 AEs in just 7 % of nivolumab treated patients vs 55 % for docetaxel. The most common immune-related toxicities were hypothyroidism, colitis, pneumonitis, nephritis, and rash, each occurring at a rate of about 4–8 % (all grades), and there were no immune-related deaths.

**Table 1:** Response rates to anti-PD-1 and overall survival in NSCLC by study

Study	Histology	Treatment	# patients	ORR (%)	Median response duration (months)	OS (months)
Nivolumab						
Gettinger et al. [11]^*a*^	All	Nivolumab 3 mg/kg q2wks	37	24.3	17	14.9
		Nivolumab 10 mg/kg q2wks	59	20.3	19.1	9.2
Rizvi et al. [13] (CHECKMATE 063)	Squamous	Nivolumab 3 mg/kg q2wks	117	14.5	Not reached	8.2
Brahmer et al. [14] (CHECKMATE 017)	Squamous	Nivolumab 3 mg/kg q2wks	135	20	Not reached	9.2
		Docetaxel 75 mg/m^2^ q3wks	137	9	8.4	6
Borghaei et al. [15] (CHECKMATE 057)	Non-squamous	Nivolumab 3 mg/kg q2wks	292	19	17.2	12.2
		Docetaxel 75 mg/m^2^ q3wks	290	12	5.6	9.4
Bauer et al. [55]	All	Nivolumab 3 mg/kg q 2wks	51	13.7	Not reported	Not reported
Pembrolizumab						
Garon et al. [16] (KEYNOTE 001)	All	Pembrolizumab 2 mg/kg q3wks	6	33.3	12.5	9.3 (prior therapy)
		Pembrolizumab 10 mg/kg q3wks	287	19.2		
						16.2 (no prior therapy)
		Pembrolizumab 10 mg/kg q2wks	202	19.3		
Herbst et al. [17] (KEYNOTE 010)	All	Pembrolizumab 2 mg/kg q3wks	344	18	Not reached	10.4
		Pembrolizumab 10 mg/kg q3wks	346	18.5	Not reached	12.7
		Docetaxel 75 mg/m^2^ q3wks	343	9.3	6	8.5

^a^Cohorts receiving doses lower than 3 mg/kg were omitted from the table due to low response rates

CHECKMATE 057 enrolled exclusively patients with non-squamous NSCLC and patients were randomized to docetaxel or nivolumab [15]. Again, all study outcomes favored nivolumab including OS (12.2 vs 9.4 months), ORR (19 % vs 12 %), and safety (grade 3 or 4 treatment related AEs in 10 % vs 54 %) with similar rates of immune-related toxicities to CHECKMATE 017. In the subgroup analysis, no survival advantage was observed for never smokers nor for those with activating EGFR mutations. Patients receiving third line therapy or with brain metastases also did not benefit from nivolumab over docetaxel, but this was probably more reflective of an underlying aggressive or advanced disease variant than of a differential treatment effect.

### Pembrolizumab

**Fig. 1:**
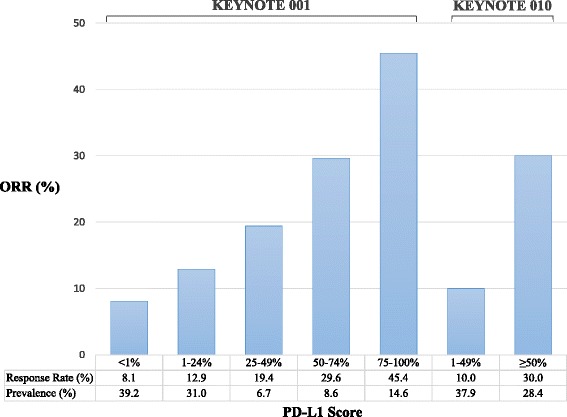
PD-L1 proportion scores and their relationship to objective response rates to pembrolizumab in KEYNOTE 001 and KEYNOTE 010

Two clinical trials have studied pembrolizumab in patients with advanced NSCLC. KEYNOTE 001 was a large phase I trial with a NSCLC expansion cohort that enrolled 495 patients who were treated with pembrolizumab 2 mg/kg or 10 mg/kg every 2–3 weeks [16]. The trial included 101 patients who had never received systemic therapy. Objective responses were seen in 19.4 % of patients, including 24.8 % of chemotherapy naïve patients, while another 21.8 % of patients achieved stable disease. Treatment was effective at all tested doses and schedules, thus an every 3 week schedule was chosen for the phase III study. Responses were more common in former or current smokers compared to non-smokers (22.5 % vs 10.3 %). Treatment-related adverse events were common (70.9 %), and 9.5 % of patients had grade 3–5 toxicities. Immune-related toxicities like hypothyroidism (6.9 %) and pneumonitis (3.6 %) were mostly manageable though one patient died of severe pneumonitis. Fresh tumor biopsies were required for PD-L1 staining by IHC, and observed response rates were higher with increasing percentages of tumor PD-L1 expression (Fig. 1). Responses were durable, lasting a median of 12.5 months (range 1–23.3 months). Median OS was 9.3 months for previously treated patients and 16.2 months for chemotherapy naïve patients.

KEYNOTE 010 was a randomized phase III trial analogous to CHECKMATE 017 and 057, comparing pembrolizumab at two doses, 2 mg/kg and 10 mg/kg every 3 weeks, to docetaxel in 1034 patients [17]. The study enrolled only patients with at least 1 % PD-L1 positive staining, with the last 593 patients stratified by PD-L1 positivity using a 50 % cutoff. Patients treated with either dose of pembrolizumab had a higher median OS than with docetaxel (10 mg/kg: 12.7 months vs 8.5 months; HR 0.61, *p* < 0.0001; 2 mg/kg: 10.4 months, HR 0.71, *p* = 0.0008). There was a non-significant trend toward longer survival in the 10 mg/kg cohort compared with the 2 mg/kg cohort. When stratified by PD-L1 positivity, defined as a proportion score (PS) ≥50 % staining of the tumor, the survival benefits were more pronounced (HR 0.50 and 0.54 for 10 mg/kg and 2 mg/kg cohorts, respectively), with median survivals of 17.3 and 14.9 months. In the subgroup analysis, both squamous and non-squamous histology favored pembrolizumab treatment, consistent with results from the nivolumab clinical trials. Similarly, EGFR mutated tumors seemed to have no survival advantage with pembrolizumab over docetaxel, though the number of patients (*n* = 86) was small. Pembrolizumab was more tolerable than docetaxel, with fewer grade 3–5 AEs (14 % vs 35 %) and fewer drug discontinuations (4.7 % vs 10 %). There were six (0.9 %) pembrolizumab-attributed deaths, three due to pneumonitis, two pneumonia, and one myocardial infarction, and five (1.6 %) docetaxel-attributed deaths. Based on the unprecedented survival achieved in the PD-L1+ population, the FDA approved pembrolizumab for second line therapy but only for tumors with a PD-L1 PS ≥50 %.

### Companion PD-L1 testing

#### Nivolumab/Dako IHC 28–8 pharmDx

In the multi-tumor phase I trial, a non-commercial anti-PD-L1 murine monoclonal antibody (5H1) was used to measure PD-L1 expression by IHC in tumor biopsies [9, 18]. Each stained tumor section was scored by the degree of membranous staining on tumor cells, with a minimum requirement of 100 evaluable tumor cells. The “positive” threshold was set at 5 % of cells based on test performance characteristics. There appeared to be differential response rates, with 36 % of PD-L1 positive vs 0 PD-L1 negative tumors experiencing a treatment response [10]. The antibody used in this assay was later abandoned in favor of commercial assay developed by Dako using clone 28–8, a rabbit anti-human PD-L1 (see Table 2). Two of the four subsequent publications using this assay in NSCLC have demonstrated an apparent value for this PD-L1 test. In the smaller CHECKMATE 063, a single arm study in squamous NSCLC, 24 % of PD-L1 positive patients (*n* = 25) and 14 % of negative patients (*n* = 51) had an objective response. In CHECKMATE 057, PD-L1 positivity at ≥5 % strongly correlated with objective response (34 % vs 14 % for PD-L1 negative) as well as predicted an OS benefit compared with docetaxel (PD-L1+ HR 0.43 vs PD-L1- HR 1.01; *p* < 0.001) [15]. Meanwhile, in CHECKMATE 017, PD-L1 positivity at any cutoff was not significantly prognostic nor predictive of benefit in squamous histology [14]. However, the power of this study was limited by a smaller sample size than CHECKMATE 057, and a closer inspection of the study outcomes suggests not only trends toward improved ORR and OS for PD-L1 positive patients, but also nearly double the number of patients in the “tail” of the PD-L1 positive progression-free survival (PFS) curve suggesting a higher likelihood of long term benefit. Most importantly, in all studies a significant proportion of PD-L1 negative patients clearly benefitted from treatment with nivolumab. Similar observations have been made in the melanoma clinical trials using the same assay (see Table 3). As a consequence, the FDA label for nivolumab did not specify any threshold PD-L1 positivity, in fact it did not require PD-L1 testing at all and the Dako 28–8 assay was labelled as “complementary.”

**Table 2:** Companion PD-L1 Assays in Development for PD-1/PD-L1 Inhibitors

Drug	Drug target	Companion antibody clone	Developer	Definition of positive test
*Nivolumab*	PD-1	28-8	Dako	≥5 % membranous staining of tumor cells (minimum 100 cells evaluated)
			Bristol-Meyers Squibb	
*Pembrolizumab*	PD-1	22C3	Dako	≥1%^a^ membranous staining of tumor cells or immune cells that are intercalating or at the tumor interface
			Merck	
*Atezolizumab (MPDL3280A)*	PD-L1	SP142	Ventana	Each specimen assigned a score based on both tumor and immune cell PD-L1:
			Genentech/Roche	
				TC3/IC3 PD-L1 ≥ 50 %
				TC2/IC2 PD-L1 5-49 %
				TC1/IC1 PD-L1 1-4 %
				TC0/IC0 PD-L1 < 1 %
*Durvalumab (MEDI4736)*	PD-L1	SP263	Ventana	≥25 % membranous staining of tumor cells
			MedImmune/AstraZeneca	

^a^The FDA indication in NSCLC for pembrolizumab requires PS ≥50 %

**Table 3:** Response rates to anti-PD-1/PD-L1 antibodies in NSCLC and selected malignancies according to PD-L1 positivity

Study	Antibody	Tumor type	PD-L1 cutoff	*N*	Response (%)
Nivolumab					
Topalian et al. [10]	5H1	Multiple	≥5 %	25	36
			<5 %	17	0
Gettinger et al. [11]	28-8	NSCLC	≥5 %	33	15
			<5 %	35	14
Rizvi et al. [13]	28-8	Squamous NSCLC	≥5 %	25	24
			<5 %	51	14
Brahmer et al. [14]	28-8	Squamous NSCLC	≥5 %	42	21
			<5 %	75	15
Borghaei et al. [15]	28-8	Non-squamous NSCLC	≥5 %	95	34
			<5 %	136	14
Hodi et al. [56]	28-8	Melanoma	≥5 %	18	44
			<5 %	23	13
Robert et al. [57]	28-8	Melanoma	≥5 %	74	53
			<5 % or indet	136	33
Weber et al. [58]	28-8	Melanoma (ipilimumab refractory)	≥5 %	55	44
			<5 %	64	20
Larkin et al. [59]	28-8	Melanoma	≥5 %	80	58
			<5 %	208	41
Pembrolizumab					
Garon et al. [16]	22C3	NSCLC	≥50 %	119	41
			<50 %	237	13
Herbst et al. [17]	22C3	NSCLC	≥50 %	290	30
			1–49 %	400	10
Kefford et al. [60]	22C3	Melanoma	≥1 %	55	51
			<1 %	16	6
Puzanov et al. [61]	22C3	Melanoma (ipilimumab refractory)	≥1 %	193	26
			<1 %	93	15
Robert et al. [62]	22C3	Melanoma	≥1 %	896	NR (PFS HR 0.53)
			<1 %	197	NR (PFS HR 0.67–0.76)
Atezolizumab (MPDL3280A)					
Spigel et al. [63]	SP142	NSCLC	TC or IC ≥50 %	53	26
			TC or IC 5–49 %	84	14
Spira et al. [20, 64]	SP142	NSCLC	TC or IC ≥50 %	24	38
			TC or IC 1–49 %	69	12
			TC and IC <1 %	51	8
Besse et al. [65]	SP142	NSCLC	TC or IC ≥50 %	302	26
			TC or IC 5–49 %	357	10
Powles et al. [66]	SP142	Urothelial	TC or IC ≥5 %	30	43
			TC and IC <5 %	35	11
Rosenberg et al. [67]	SP142	Urothelial	TC or IC ≥5 %	100	26
			TC and IC 1–5 %	107	11
			TC and IC <1 %	103	8
Herbst et al. [21]	SP142	Multiple	TC or IC ≥50 %	33	46
			TC or IC 1–49 %	57	19
			TC and IC <1 %	60	13
Durvalumab (MEDI4736)					
Rizvi et al. [68]	SP263	NSCLC	≥25 %	84	27
			<25 %	92	5
Segal et al. [69]	SP263	HNSCC	≥25 %	22	18
			<25 %	37	8
Avelumab (MSB0010718C)					
Gulley et al. [70]	?	NSCLC	≥5 %	122	15
			<5 %	20	10
Apolo et al. [71]	?	Urothelial	≥5 %	10	40
			<5 %	22	9

#### Pembrolizumab/Dako IHC 22C3 pharmDx

The pembrolizumab companion PD-L1 assay, also commercially available from Dako, uses a distinct antibody clone, 22C3 (mouse anti-human PD-L1). This automated assay is performed on formalin-fixed, paraffin-embedded (FFPE) tissue using similar conditions for heat-based antigen retrieval and staining as the 28–8 assay. After staining is completed, the percentage of membranous PD-L1 staining of neoplastic *or intercalating immune cells* is counted manually and the PS is reported as a percentage (see Table 2).

In KEYNOTE 001 for NSCLC, it was evident early in the trial that increasing efficacy correlated with PD-L1 positivity by this IHC assay [19]. Following enrollment of 51 patients, the study was modified to include only patients with at least 1 % PD-L1 positivity [16]. The investigators also noted that when archival tissue over 6 months old was used for testing, the PD-L1 protein had deteriorated resulting in unreliable staining. To identify an optimal cutoff for PD-L1 positivity, a training cohort of 61 tumors was stained for PD-L1 and a threshold PS ≥ 50 % was established as the positive threshold. Among the total screened patients, the prevalence of PD-L1 PS ≥50 % was 23.2 %, while another 37.6 % had a PS between 1 and 49 %. Patients with activating EGFR mutations or ALK rearrangement were equally as likely to have high PD-L1 expression as non-mutated tumors, though the total patients with these mutations was low.

At the time of analysis, both PFS and OS were considerably longer for the group with a PD-L1 PS ≥50 % (~40 and 65 % at 1 year, respectively), while PFS and OS were similar for the groups with a PS ≤1 % or 1–49 % (~10 and 40 % at 1 year). The duration of response, however, was no different between groups, suggesting that even patients with PD-L1 “negative” tumors could attain a durable, meaningful benefit albeit at a much lower frequency than the PD-L1 “positive” tumors.

In KEYNOTE 010 the same assay was used with a threshold for PD-L1 high (PS ≥50 %), intermediate (PS 1–49 %), or low (PS ≤1 %) tumors, roughly a third of patients fell into each category and those with PS ≤1 % were excluded from the trial. As noted earlier in this review, patients with a higher PS were much more likely to have an objective response to pembrolizumab (30 %), however responses were still observed in 10 % of those with a PS 1–49 % and the OS subgroup analysis still favored pembrolizumab over docetaxel (HR 0.76, 95 % CI 0.60–0.96). Interestingly, this group with intermediate PD-L1 expression did not have a PFS advantage over docetaxel (HR 1.04), a potential indicator that atypical immunologic anti-tumor responses are more common in this subset.

### Other antibodies in development

Several anti-PD-1 (pidilizumab/CT-011, REGN2810) and anti-PD-L1 antibodies (durvalumab/MEDI4736, atezolizumab/MPDL3280A, avelumab/MSB0010718C, BMS-936559) are in various stages of clinical development for NSCLC and other cancers. Like nivolumab and pembrolizumab, these agents are designed to block the interaction of PD-1 with PD-L1 and most have been modified to have no Fc-mediated antibody dependent cellular cytotoxicity.

A phase II randomized trial (POPLAR) with atezolizumab was recently published. In this trial, 287 patients with previously treated advanced or metastatic NSCLC were randomized 1:1 to docetaxel or atezolizumab (given at a flat dose of 1200 mg IV every 3 weeks) [20]. Overall survival, the primary endpoint, was improved in the atezolizumab arm by nearly 3 months (median OS 12.6 months vs 9.7 months; HR 0.73, *p* = 0.04), while safety was similar to other anti-PD-1 agents (11 % with treatment related grade 3 or 4 AEs). Responses lasted a median of 14.3 months (vs 7.2 months for docetaxel), while neither the ORR nor PFS were higher in the atezolizumab arm, confirming that traditional radiographic criteria are imprecise measures of benefit from immunotherapy.

Importantly, enrollment was stratified by PD-L1 expression using a novel IHC assay (Ventana SP142, Table 3) in which PD-L1 positivity was categorized according to the expressing cell type (tumor cell [TC] or immune cell [IC]) and then scored along a gradient (<1 % [TC0 or IC0], 1–4 % [TC1 or IC1], 5–49 % [TC2 or IC2], and ≥50 % (TC3 or IC3]). Treatment with atezolizumab was favored in all but the least PD-L1 positive tumors (TC0 and IC0; HR 1.04). Other biomarkers were explored, including IHC expression of PD-L2, B7.1 (an alternative receptor for PD-L1), and PD-1 as well as an expression panel of T-effector and interferon-γ associated genes, all of which were predictive of a survival benefit from atezolizumab.

### PD-L1 testing limitations

As outlined, considerable effort has been invested to develop quantifiable, reproducible PD-L1 assays to predict which patients should receive immune checkpoint inhibitors. The commercial complementary PD-L1 diagnostic test for nivolumab (Dako 28–8 pharmDx) and companion test for pembrolizumab (Dako 22C3 pharmDx) are now FDA approved for use in NSCLC, while the complementary test for atezolizumab (Ventana SP142) is approved for urothelial carcinoma. The performance characteristics of the companion tests for atezolizumab and durvalumab (Ventana SP263) are still being assessed in NSCLC (Table 3). It is expected that each of these drugs and assays will ultimately become available to practicing oncologists.

Still, the performance of these IHC-based assays has been somewhat disappointing, as most “positive” cutoffs would exclude a considerable number of responders in the range of 10–20 %. Therein lies the first drawback to PD-L1 testing, which is, how is PD-L1 “positivity” defined? PD-L1 can be expressed by both tumor and inflammatory cells within the tumor microenvironment, though the relative importance of either is unclear [21]. There is no consensus to the relevance of geographic patterns of expression (eg. proximity of PD-L1 to immune infiltrating lymphocytes, membranous vs cytoplasmic) and quantitative cutoffs have been variably described. Based on the findings in KEYNOTE 001 (Fig. 1), it may make more sense to consider PD-L1 expression as a continuous measures rather than a binary “positive” or “negative.” Along these lines, currently published tissue studies have found PD-L1 positivity to indicate favorable, unfavorable, or have no relationship to prognosis, as well as variable correlations with histology and mutation status in NSCLC and other tumor types [22–32].

Technical aspects of the assays are also an important source of inconsistency. This topic was reviewed in detail recently [33, 34]. The quality of commercially available antibodies is a major concern with considerable variability in staining intensity and patterns found between antibodies [23]. Until recently, no information was available to compare the companion diagnostics in development by Dako and Ventana (Table 2). Assay variables pertaining to tissue fixation, storage, and antigen retrieval can result in PD-L1 degradation and these variables are not standardized. Importantly, the KEYNOTE 010 trial confirmed that archival tissue (as opposed to fresh tissue) can be used for PD-L1 staining and this has been observed in other trials [17, 35]. But the lack of methodologic transparency and standardization across platforms may partially explain why PD-L1 positivity has had such widely varying clinical significance, not only in NSCLC but also in other tumor types. Table 3 lists response rates for anti-PD-1 and anti-PD-L1 treatments according to companion PD-L1 “positivity” in NSCLC and selected other cancers trials.

To address these concerns, a consortium of drug manufacturers and representatives from Dako and Ventana, organized in part through a joint effort of the FDA, AACR, ASCO, and the International Association for the Study of Lung Cancer (IASLC), was formed with the task of creating a resource to compare the performances of the four major PD-L1 companion assays. The ultimate goal of this collaboration is to establish cross-platform standards for PD-L1 positivity analogous to those for ER, PR, and HER2 testing. Results of the pilot phase of this “Blueprint project” were presented at the AACR Annual Meeting in 2016, and included 39 lung cases reviewed by 3 pathologists [36]. Tumor cell PD-L1 staining was similar for the Ventana SP263 and two Dako assays, with less tumor staining by the Ventana SP142 assay. Interobserver variability was high when quantifying immune cell positivity but not tumor cell positivity. The performance of these assays (using their respective PD-L1 staining thresholds) varied substantially, with the Ventana SP142 assay labeling the highest number of samples as positive (79 %) and the Ventana SP263 labeling the fewest as positive (53 %). Both Dako assays (28–8 and 22C3) performed nearly identically with an intermediate rate of positivity, and there was incomplete overlap between all of the assays. These important preliminary observations highlight the potential for false positive and negative results based solely on the assay chosen. Additional predictive information, larger sample sizes, and potentially adjustments to the cutoffs will be required before any conclusions can be made about the merits of each assay.

### Additional challenges to biomarker development

There are other biological limitations to PD-L1 detection in tumor biopsies. Most traditional cancer biomarkers evaluate fixed elements that do not vary tremendously with time such as gene mutations or proteins directly involved in cellular growth signaling or replication. Examples include estrogen and progesterone receptor expression, HER2 gene amplification, or mutations in EGFR, KRAS, etc. Alternatively, the PD-L1 gene is not typically mutated or amplified and its expression is dynamic both in space and over time in response to a constantly evolving immune response. There is concern that sampling error could result in false negative tests, though some recent case series have suggested a reasonable concordance between both synchronous (same time but different location) and metachronous (different time) specimen in the range of 75–90 % [37–40]. PD-L1 expression is also affected by concurrent or prior treatments, including radiation or chemotherapy, which may have been administered after a biopsy was obtained [41–44].

Alternative biomarker approaches have focused on quantifying and qualifying tumor infiltrating lymphocytes (TILs) or on identifying tumor neoantigens, which are fragments of mutated proteins displayed in the major histocompatibility complexes (MHC) of tumor cells and which are probably critical to the anti-tumor immune response. Several studies now have found that the mutational load, as well as the number of predicted neoantigens (according to computerized algorithms which account for MHC binding), are better predictors of response to checkpoint inhibition than PD-L1 IHC, TILs, or clinical variables [45–47]. While efforts are underway to better understand and identify these neoantigens through exome sequencing, surrogate measures of the mutation burden such as chronic carcinogen exposure (eg. tobacco, ultraviolet light) and defects in DNA repair mechanisms (microsatellite instability/mismatch repair defects, BRCA and POLE mutations) have emerged as clinically useful biomarkers [46–48].

## Conclusions

The recent FDA approvals of nivolumab and pembrolizumab represent the earliest phase of a major paradigm shift in treating NSCLC. So far, it appears that both anti-PD-1 antibodies, as well as the therapeutic anti-PD-L1 antibodies in development, will have comparable efficacy and toxicity, with responses in approximately 15–20 % of unselected NSCLC patients and serious autoimmune toxicities in 5–10 % of patients. In the absence of head-to-head to comparisons or clear biological differences between these agents, it is not possible to recommend one treatment over another.

Due to the low toxicity of checkpoint inhibitors and the theorized synergy with other treatment modalities [49, 50], combination clinical trials of anti-PD-1 antibodies with chemotherapy, radiation, and other immunotherapies are ongoing in the metastatic setting. The most mature of these have combined anti-PD-1 or anti-PD-L1 antibodies with anti-CTLA-4 antibodies with impressive results at the cost of increased toxicity [51–54]. This strategy has been effective in the treatment of melanoma. It also remains to be proven that these agents are as effective in the first-line setting, though in the treatment naive cohort in KEYNOTE 001 pembrolizumab appeared equally efficacious and rates of PD-L1 positivity were comparable to previously treated patients (~23 % with PD-L1 PS ≥50 % in either group).

In the current clinical realm of FDA-approved immunotherapies, the role of PD-L1 testing is largely prognostic for patients beginning treatment with either nivolumab or pembrolizumab given that none of the assays can conclusively identify non-benefiting patients. In light of the preliminary results from Blueprint, it is not possible to recommend one assay over another. From a practical standpoint some clinicians may choose the 22C3-based assay to have optional access to pembrolizumab in the event of a positive assay. Meanwhile many laboratories have begun reporting both tumor and immune cell positivity analogous to the atezolizumab companion assay by Ventana, however the meaning of these separate scores is still very much uncertain. As more treatment options and combinations become available, measures of immune activation including PD-L1 expression will clearly become more relevant, particularly as the clinician factors the line of therapy and weighs the potential benefit and toxicity of combination immunotherapy in PD-L1 positive versus negative tumors. Specifically, it remains to be shown whether PD-L1 negative patients should be treated concurrently with an anti-CTLA-4 antibody (such as ipilimumab or tremelimumab) as appears to be the evolving strategy for melanoma. It seems PD-L1 expression represents one lens and sensitivity and specificity is likely to be more robust as multiple lenses are used in the analyses.

## Abbreviations

AE, adverse event; FFPE, formalin-fixed, paraffin-embedded; GI, gastrointestinal; HR, hazard ratio; IC, immune cell; IHC, immunohistochemistry; MHC, major histocompatibility complexes; NSCLC, non-small cell lung cancer; OR, odds ratio; ORR, overall response rate; OS, overall survival; PD-1, programmed cell death protein 1; PD-L1, programmed death ligand 1; PFS, progression-free survival; PS, proportion score; RECIST, response evaluation criteria in solid tumors; TC, tumor cell; TILs, tumor infiltrating lymphocytes
